# New race and ethnicity standards: elucidating health disparities in diabetes

**DOI:** 10.1186/1741-7015-10-42

**Published:** 2012-04-30

**Authors:** Peter T Katzmarzyk, Amanda E Staiano

**Affiliations:** 1 Population Science, Pennington Biomedical Research Center, 6400 Perkins Road, Baton Rouge, LA 70808, USA

**Keywords:** diabetes, race, ethnicity, health disparities, chronic disease, physical inactivity, dietary patterns, obesity

## Abstract

The concepts of race and ethnicity are useful for understanding the distribution of disease in the population and for identifying at-risk groups for prevention and treatment efforts. The U.S. Department of Health and Human Services recently updated the race and ethnicity classifications in order to more effectively monitor health disparities. Differences in chronic disease mortality rates are contributing to race and ethnic health disparities in life expectancy in the United States. The prevalence of diabetes is higher in African Americans and Hispanics compared to white Americans, and parallel trends are seen in diabetes risk factors, including physical inactivity, dietary patterns, and obesity. Further research is required to determine the extent to which the observed differences in diabetes prevalence are attributable to differences in lifestyle versus other characteristics across race and ethnic groups.

## Introduction

'Distinctive racial or ethnic patterns of disease can be profitably applied by the public health official to the detection and prevention of disease and by the clinician in diagnosis and treatment.' Albert Damon, 1969 [1] p.79

Overall life expectancy at birth has increased steadily in the United States from 47 years in 1900 to 78 years in 2007 [2]. The increase in life expectancy has resulted from improvements in public health (for example sanitation, infectious disease control, food security) and the prevention and treatment of medical conditions. However, there remain significant racial gaps [2,3]. In 2007, white Americans could expect to live 4.8 years longer than African Americans under current mortality patterns [2].

A major public health goal in the United States is to achieve health equity, eliminate disparities, and improve the health of all groups by 2020 [4]. A total of 51% and 64% of the racial gap in life expectancy in men and women, respectively, is attributable to differences in mortality rates from diabetes, cardiovascular disease, and cancer [3]. Indeed, diabetes is the eighth and fourth leading cause of death among white and African Americans, respectively [5]. Thus, efforts to reduce racial disparities in health should focus on understanding and reducing differences in the rates of chronic diseases such as diabetes.

There is considerable regional variation in the prevalence of diabetes at the global level; however, the overall prevalence is high and continues to increase [6]. Regional differences in the prevalence of diabetes are undoubtedly the result of complex interactions among socioeconomic forces, lifestyle factors, and genetic predisposition. Given that race and ethnic disparities are often viewed within a country-specific context, the focus of this discussion is on the United States.

### Concepts of race and ethnicity

Race and ethnicity are interrelated concepts that have a long history in the fields of human biology and public health [1,7]. Although the terms are often used interchangeably in the literature and there are no widely accepted definitions, race and ethnicity tend to have distinct meanings. Race is typically used to refer to groups that share biological similarities, whereas ethnicity refers to shared cultural similarities. In many cases, race and ethnic groups may overlap considerably; however, race and ethnicity are useful concepts when attempting to understand differential health risks and health disparities [8,9].

The Institute of Medicine [10] reported that inadequate data on race and ethnicity lowered the likelihood of effective actions to address health disparities. In response, the Department of Health and Human Services recently updated standards to more consistently measure race and ethnicity and thereby improve the ability to monitor improvements in health disparities (Table 1) [11]. These new race and ethnic categories expand upon the current Office of Management and Budget (OMB) classifications that are often used in research [12]. The use of the new categories will allow for the more precise identification of health risks in specific race and ethnic groups, and could translate into more individualized treatment regimens for the prevention and management of diabetes in the future.

**Table 1:** New categories of race/ethnicity established by the U.S. Department of Health and Human Services [11]

Race	Ethnicity
• White	• Not of Hispanic, Latino/a, or Spanish origin
• Black or African American	• Hispanic Mexican, Mexican American, or Chicano/a
• American Indian or Alaska Native	• Hispanic Puerto Rican
• Asian Indian	• Hispanic Cuban
• Chinese	• Other Hispanic, Latino, or Spanish origin
• Filipino	
• Japanese	
• Korean	
• Vietnamese	
• Other Asian	
• Native Hawaiian	
• Guamanian or Chamorro	
• Samoan	
• Other Pacific Islander	

### Racial and ethnic differences in diabetes prevalence

**Figure 1:**
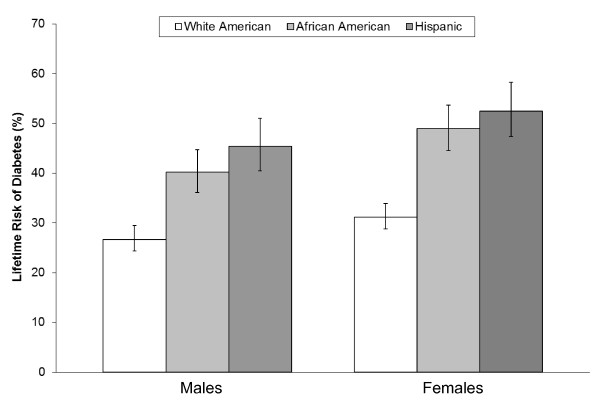
**Lifetime risk (%) of developing diabetes in the United States among individuals born in 2000**. Adapted from results presented in Narayan *et al*. [15]. Error bars indicate 95% confidence intervals.

Diabetes affects an estimated 20.4 million adults (9.6%) in the U.S., of which 19% are undiagnosed [13]. However, the age-adjusted prevalence of total diabetes (diagnosed and undiagnosed) differs by race and ethnicity, as African Americans (14.9%) and Mexican Americans (15.6%) had approximately double the prevalence as white Americans (7.6%) [13] in the 2003-2006 U.S. National Health and Nutrition Examination Survey (NHANES). Ethnic disparities are also evident in the number of medically diagnosed diabetes cases identified in the 2008 National Health Interview Survey, in which the age-adjusted prevalence of diagnosed diabetes for adults was 11.0% in African Americans, 10.7% in Hispanics, 8.2% in Asians, and 7.0% in whites [14]. The higher prevalence of diabetes among African Americans and Hispanics translates into a higher lifetime risk of developing diabetes than in white Americans (Figure 1) [15].

Race and ethnic differences in diabetes prevalence persist across different subgroups of the population. For example, among adults aged 65 years and over, 32.7% of African Americans, 31.8% of Mexican Americans, and 18.8% of white Americans have diagnosed or undiagnosed diabetes [13]. Few cases of diabetes are reported in youth; however, the prevalence of type 2 diabetes is two- to three-fold higher among American-Indian and African American youth compared to Asian/Pacific Islander and Hispanic youth, and nine-fold higher than in white youth [16]. Among immigrants to the U.S., diabetes prevalence increases as length of residence increases, independent of age or body mass index [17]. There is also evidence that risk factors such as physical inactivity and obesity differ according to length of residence in other Western countries such as Canada [18,19].

### Diabetes, ethnicity, and race: the role of lifestyle factors and socioeconomic status

#### Physical inactivity

Physical inactivity is an important risk factor for the development of diabetes [20]. Based on data from the 2005 U.S. Behavioral Risk Factor Surveillance System (BRFSS), African Americans and Hispanics had lower levels of leisure-time physical activity than white Americans [21]. These racial and ethnic differences in self-reported leisure-time physical activity appear to begin in adolescence [22]. On the other hand, based on objectively-measured (accelerometry) data from NHANES 2003-2004, Mexican American adults had higher physical activity levels compared to African Americans and white Americans [23]. Further, Mexican Americans were also less sedentary (< 100 accelerometer activity counts/min) than white or African Americans across the lifespan, while white and African Americans had similar levels of sedentary behavior [24]. The discrepant race and ethnic differences observed for self-reported versus objective methods are difficult to explain. The differences could be due to cultural influences in the reporting of physical activity, or the different aspects of human movement captured by the two methods: the accelerometry used in NHANES captures total ambulatory physical activity, including leisure-time, occupational, and domestic (such as chores) domains, whereas the BRFSS questionnaire captures information on leisure-time physical activity only.

#### Dietary patterns

A healthy dietary pattern has been linked to a reduced risk of developing diabetes [25]. For example, the consumption of red meat is associated with a higher risk of diabetes [26], and the intake of leafy green vegetables is associated with a lower risk [27]. Among adults from the Lower Mississippi Delta region of the U.S, whites had a higher Healthy Eating Index (HEI) as well as higher component scores for grains, vegetables, milk, and variety than African Americans [28]. Results from successive waves of NHANES (from 1971 to 2002) indicate that differences in dietary patterns between white and African Americans, such as the higher energy density of foods consumed among African Americans, have persisted over time [29]. These results suggest that race differences in dietary patterns may be significant; however, more research is required to better delineate the extent of the differences and their potential impact on health.

#### Obesity

**Figure 2:**
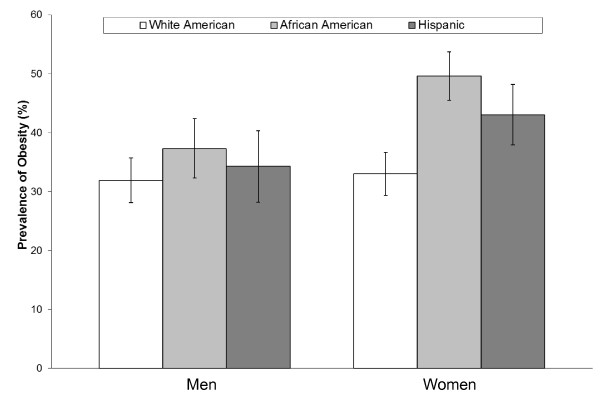
**Age-adjusted prevalence of obesity among U.S. men and women adapted from results published from the 2007-08 U.S. National Health and Nutrition Examination Survey **[33]. Error bars indicate 95% confidence intervals.

Obese individuals have 20 to 50 times greater risk of developing diabetes than people who are normal weight [30,31]. The estimated lifetime risk of developing diabetes from the age of 18 years in the U.S. increases from 19.8% and 17.1% for normal weight (body mass index (BMI) 18.5-24.9 kg/m^2^) men and women to 70.3% and 74.4% for men and women with a BMI ≥35 kg/m^2^, respectively [32]. The prevalence of obesity differs across race and ethnic groups in the U.S. The most recent data from the 2007-2008 NHANES indicates that the age-adjusted prevalence of obesity (BMI ≥30 kg/m^2^) was 32.4% in whites, 37.9% in Hispanics, and 44.1% in African Americans, and these differences are consistent in men and women (Figure 2) [33].

The degree to which race and ethnic groups differ in absolute risk for incident diabetes at a given level of body fatness has not been clearly delineated. After controlling for body weight, diabetes prevalence was more than twice as high among African Americans and Latinos compared to white Americans in a U.S. cohort [34]. Few studies have employed prospective designs to study racial differences in the relationship between obesity and diabetes. Data from the Atherosclerosis Risk in Communities (ARIC) Study indicated that the incidence of diabetes over a 9-year period was higher at all levels of BMI in African Americans compared with white adults [35]. This is in contrast to results from the NHANES Epidemiologic Follow-up Study in which the 20-year incidence of diabetes was higher in African Americans than white Americans at low BMI values, but equivalent at higher BMI values [36]. More research is required to understand the differential risks for diabetes related to obesity across race and ethnic groups.

#### Socioeconomic status

Race and ethnic disparities in diabetes prevalence may be confounded by socioeconomic inequalities. On average, African Americans and Hispanics tend to be poorer and less educated [37] and less likely to have health insurance [38], compared to white Americans. In the U.S. National Health Interview Survey, diabetes prevalence was highest among individuals with low educational attainment and those below the federal poverty line [14]. Disparity in diabetes prevalence due to income and education increased from 2004 to 2008, whereas race/ethnic disparities in diabetes prevalence and incidence did not change [14]. There was little race/ethnic difference in diabetes prevalence among a cohort of African American and white adults of similar low socioeconomic status from the southern U.S., except a moderately higher rate in African American versus white women [39]. Because socioeconomic status is a potentially modifiable factor, interventions to prevent diabetes could focus on improving social circumstances and access to care among the less educated and impoverished [40].

## Conclusions

The concepts of race and ethnicity are useful in understanding the distribution of diabetes and related risk factors in the population. Data from representative surveys from the U.S. have demonstrated significant race and ethnic differences in the prevalence of diabetes and parallel differences in lifestyle risk factors. This mounting evidence for race and ethnic differences may indeed prove profitable both in understanding the epidemiology of diabetes and in targeting at-risk groups for prevention and treatment efforts. However, further research is required to determine the extent to which the disparities in diabetes risk are attributable to differences in lifestyle versus other characteristics that cluster within race and ethnic groups, such as differences in genetics or metabolism.

The new, more precise categories of race/ethnicity will allow investigators and clinicians to better understand disparities and to create individualized or group-specific treatment plans that target the individuals most at risk for the development of diabetes and related complications. Although the role of lifestyle factors in explaining race and ethnic differences has not been fully delineated, the adaptation of current physical activity and dietary guidelines for use in different ethnic and race groups could prove beneficial to prevention efforts. In addition, prevention and treatment efforts could target lifestyle factors that are known to be disproportionately higher or lower in specific race/ethnic groups.

## List of abbreviations

ARIC: Atherosclerosis Risk in Communities; BMI: body mass index; BRFSS: U.S. Behavioral Risk Factor Surveillance System; HEI: Healthy Eating Index; NHANES: U.S. National Health and Nutrition Examination Survey; OMB: Office of Management and Budget.

## Competing interests

The authors declare that they have no competing interests.

## Authors' contributions

PTK and AES researched the literature and drafted the manuscript. Both authors approved the final version.

## Authors' information

PTK is Associate Executive Director for Population Science, Professor and Louisiana Public Facilities Authority Endowed Chair at Pennington Biomedical Research Center. AES is a Postdoctoral Research Fellow in the Division of Population Science at Pennington Biomedical Research Center.

## Pre-publication history

The pre-publication history for this paper can be accessed here:

http://www.biomedcentral.com/1741-7015/10/42/prepub
